# Epidemiology of Human Bocavirus in the Middle East and North Africa: Systematic Review

**DOI:** 10.3390/pathogens10111456

**Published:** 2021-11-10

**Authors:** Rana Abdelqader, Hanan Hasan, Lo’ai Alanagreh

**Affiliations:** 1 Department of Basic Medical Sciences, Faculty of Applied Medical Sciences, The Hashemite University, Zarqa 13133, Jordan; 2 Department of Pathology, Microbiology and Forensic Medicine, School of Medicine, The University of Jordan, Amman 11942, Jordan; hananyalu97@gmail.com; 3 Department of Medical Laboratory Sciences, Faculty of Applied Medical Sciences, The Hashemite University, Zarqa 13133, Jordan; loai-alanagreh@hu.edu.jo

**Keywords:** human bocavirus (HBoV), MENA, epidemiology

## Abstract

The emergence of the COVID-19 pandemic highlighted the importance of studying newly emerging viruses that cause respiratory illnesses. Human bocavirus (HBoV) is one of the relatively newly discovered viruses that has been detected worldwide and causes respiratory and gastrointestinal infections, mainly in pediatric patients. However, little is known about the pathogenicity and evolution of HBoV. This systematic review was initiated to clarify the prevalence and circulating genotypes of HBoV in both respiratory and stool samples from patients of all age groups in the Middle East and North Africa (MENA) from 2005 to February 2021. We performed an electronic search through Science Direct, Scopus, PubMed, Mendeley and Cochrane Library databases. We included all studies reporting the detection rate of HBoV in the MENA region. Data were extracted, and the quality of the included articles was assessed. We included articles containing data on HBoV only or with other respiratory or gastrointestinal viral infections. Review articles, case studies, and animal and environmental studies were excluded. The final number of articles included in this study was 65 articles. The results showed that the HBoV prevalence in children was the lowest in Iran (0%) and the highest in Egypt (56.8%). In adults, the lowest and the highest prevalence were reported in Iran, with values of 0% and 6.6%, respectively. Regarding the respiratory cases, our findings revealed no significant difference between HBoV prevalence among the tested categories (*p*-value = 0.998). The present study has shown that HBoV is common in children and adults in the MENA region. This systematic review highlights the need for more data on the role of coinfection of HBoV and other viruses, for instance, SARS-CoV-2 in children with acute bronchiolitis.

## 1. Introduction

Human bocavirus (HBoV) is a parvovirus reported for the first time in 2005 [1]. Since then, an increasing number of reports have emerged indicating the common presence of the virus in the respiratory and gastrointestinal samples. HBoV is known to cause viral respiratory and gastrointestinal tract infections [1,2]. However, the pathogenicity of the virus is not fully understood [3,4]. As with other viruses that cause respiratory tract infections, HBoV can occur during any time of the year, with the highest incidence rate during winter and spring [5,6]. Although HBoV has been found in individuals of all ages, it was mainly reported in infants aged 6–24 months [4,5].

HBoV is a small non-enveloped single-stranded DNA virus with a genome size of 5300 nucleotides. The name Bocavirus was derived after the phylogenetic analysis of the HBoV genome, which showed a close relation to bovine parvovirus (BPV1) and minute virus of canines (MVC). HBoV belongs to the family *Parvoviridae*, subfamily *Parvovirinae* and genus *Bocavirus*. There are four genotypes that belong to the Bocavirus genus. The first genotype was named HBoV1, and was predominantly reported in respiratory samples [7]. The others, named HBoV2, 3 and 4, were reported in the stool samples of gastroenteritis patients [8].

**Figure 1:**
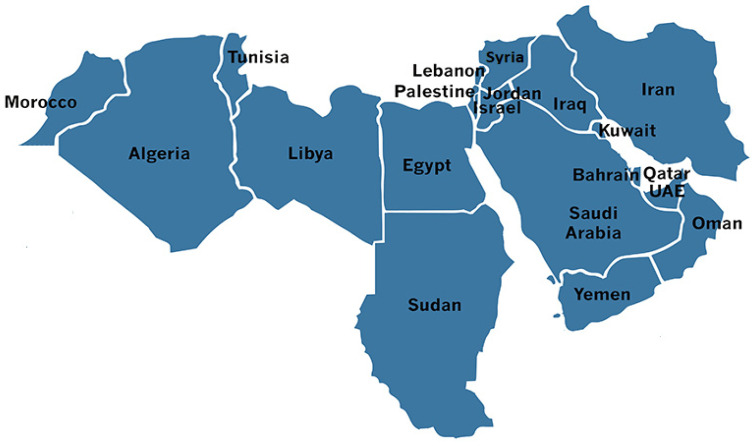
The Middle East and North Africa region (MENA) (https://istizada.com/mena-region/ (assessed on 16 January 2021).

Globally, the total prevalence of HBoV was estimated at around 6.0% [3]. Death cases due to HBoV infections have been reported [9,10,11]. However, there is no definite death rate. The Middle East and North Africa (MENA) region is a term that represents a group of twenty-one countries found in Asia (Bahrain, Iran, Iraq, Israel, Jordan, Kuwait, Lebanon, Oman, Qatar, Saudi Arabia, Syria, Turkey, United Arab Emirates, Palestine and Yemen) and Africa (Algeria, Egypt, Libya, Morocco, Sudan and Tunisia) (https://istizada.com/mena-region/, accessed on 16 January 2021) (Figure 1). Only 14 countries reported the prevalence of HBoV in the MENA region. On the other hand, a lack of reported data was noticed in several countries (Bahrain, Syria, Palestine, Yemen, Algeria, Libya and Morocco) due to lack of knowledge, awareness and attitude of physicians, wars, conflicts, civil revolutions and low scientific research output. The aim of this systematic review was to investigate the prevalence of HBoV and its distribution in the MENA region. Data included patients of all age groups, mainly children, with acute respiratory and gastrointestinal infections, including pilgrims returning from Hajj and Umrah and suffering from acquired acute respiratory tract illness (ARI).

## 2. Methods

### 2.1. Search Strategy and Selection Criteria

This systematic literature review involves all published journal articles and preprints that reported HBoV prevalence and genotypes in the Middle East and North Africa (MENA) region between 2005 and February 2021. Five databases were searched (Science Direct, Scopus, PubMed, Mendeley and Cochrane Library) by using (“boca*” OR “bocavirus” OR “boca virus”) AND (“gastro*” OR “genotype” OR “epidemiology” OR “resp*” OR “prevalence” OR “type”) AND (“The Middle East” OR “North Africa” OR “The Middle East and North Africa” OR “The Middle East & North Africa” OR “MENA” OR “Algeria” OR “Bahrain” OR “Djibouti” OR “Egypt” OR “Iran” OR “Iraq” OR “Jordan” OR “Kuwait” OR “Lebanon” OR “Libya” OR “Morocco” OR “Occupied Palestinian Territories” OR “Oman” OR “Palestine” OR “Qatar” OR “Saudi Arabia” OR “KSA” OR “Somalia” OR “Sudan” OR “Syria” OR “Tunisia” OR “UAE” OR “The United Arab Emirates” OR “Yemen”) as a search strategy. The eligible articles were screened for both the titles and abstracts. The studies involved in this systematic review were selected based on the following criteria: (1) the published articles contain data on HBoV only or with other respiratory or gastrointestinal viral infections from 2005 to February 2021, (2) the studied population in the article is patients residing in, or having acquired infection from, the MENA region. Review articles, case studies, and animal and environmental studies were excluded.

### 2.2. Data Collection and Data Adjustment 

**Figure 2:**
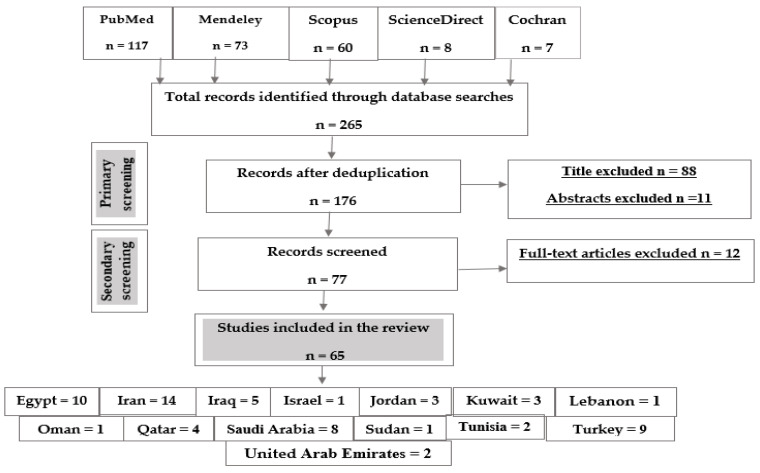
Flow chart of the study selection protocol.

Following the research strategy, a total of 265 articles were identified as follows: 117 articles from PubMed, 73 from Mendeley, 60 from Scopus, eight from Science Direct and seven articles from Cochran. The number of records after deduplication was 175, 88 articles were excluded due to their titles, 11 articles were excluded due to their abstracts and 12 articles were excluded after full-text article screening. The final number of articles included in this study was 65 articles (Figure 2).

The data collection sheet was designed to extract data from the selected articles at a 95% confidence interval. The prevalence data were extracted and arranged according to the country and year of sample collection and were reported as percentages. Data of respiratory records were compared by Fisher’s exact test, and p-values were calculated in IBM SPSS statistics version 28 by using the Chi-square test to identify associations.

The summary of individual study parameters was prepared using Microsoft Excel. A mean percentage prevalence was taken if more than one prevalence study was reported from the same country. Prevalence charts were produced for both respiratory and gastrointestinal samples.

## 3. Results

**Figure 3:**
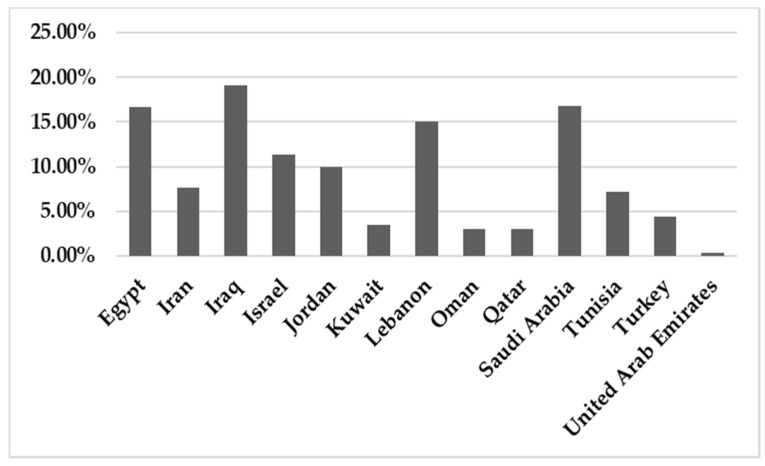
The prevalence of HBoV in respiratory samples in the MENA.

**Figure 4:**
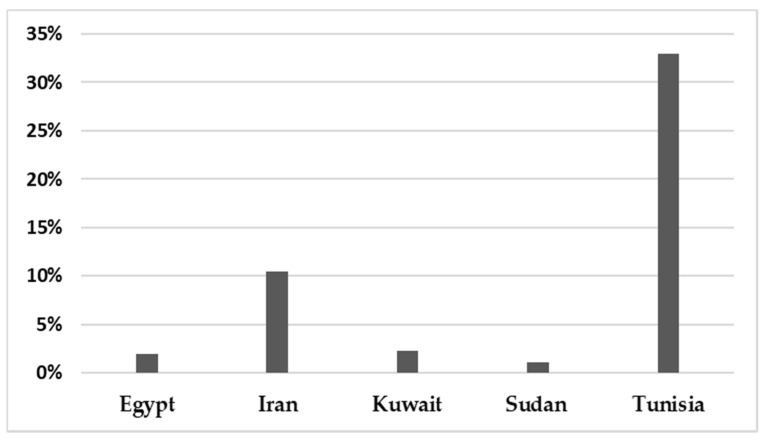
The prevalence of HBoV in gastrointestinal samples in the MENA.

In total, 142,748 patients were reported in sixty-five studies, and 5622 (3.94%) were positive for infection. All those studies reported the prevalence of HBoV in the MENA from 2005 to February 2021 (Table 1). A mean percentage prevalence was calculated for each country for both respiratory and gastrointestinal samples. The prevalence charts were constructed for both respiratory (Figure 3) and gastrointestinal samples (Figure 4).

This systematic review reports the prevalence of HBoV in the MENA region among different tested categories including various age groups (pediatric, children, adults and elderly), COVID-19 cases, pilgrims, health care providers, blood donors and patients with colorectal cancer. Concerning the respiratory cases, our findings revealed no significant differences between HBoV prevalence values among the tested categories (*p*-value = 0.998).

The study design for almost all of the included studies was a cross-sectional study that aligns with the prevalence determination. In addition, a pilot study, case-control and cohort studies were included in this systematic review. The different study designs can explain the heterogeneity of the sample size.

All included studies used valid assay procedures for the detection of HBoV. The most commonly used method is real-time polymerase chain reaction (RT-PCR). Samples from the upper (nasopharyngeal aspirates, nasopharyngeal swabs or oropharyngeal swab), middle (tracheal aspirate) and lower respiratory tract (Broncho alveolar lavage) were examined for patients with respiratory tract infection. Stool was the specimen of choice for patients with gastroenteritis. 

Surgically excised specimens were used to screen human bocavirus in colorectal cancer patients. Whole blood samples from blood donors were screened for HBoV to investigate the possibility of parenteral transmission.

## 4. Discussion

In the MENA Region, several reports studied the prevalence of HBoV among hospitalized children and adults suffering respiratory tract infections and whether HBoV was the causal agent [27,41]. At the same time, others investigated the HBoV prevalence in patients with gastroenteritis [14,24].

The results showed that the prevalence of HBoV varied from one country to another. The HBoV prevalence, in cases of respiratory tract infection in children, ranged from 0% in Iran to 56.8% in Egypt [16,30]. In adults, the highest prevalence (6.6%) was observed in Iran [27]. Few studies have focused on HBoV isolated from stool specimens to recognize the role of HBoV in gastroenteritis. Only nine studies were found in the MENA, five of them from Iran, and the others were conducted on populations in Egypt, Kuwait, Sudan and Tunisia. Among these studies, the lowest prevalence was reported in Sudan in 2018 (1.10%) [61], while the highest prevalence (33%) was reported in Tunisia [62]. Several factors affect the variations in the prevalence of the virus in these populations, including the geographical location of the country, the clinical diagnosis of the studied population, the type of sample, the method used for detection of the virus, the age group of the examined population and the outbreak season of the virus.

Abdel-Moneim et al. (2016) used newly developed primers to increase the sensitivity of the PCR test for HBoV detection. Using these novel primers, the prevalence of HBoV was 56.8%, which significantly differs from previous and further studies conducted in Egypt, which found prevalence values of 22%, 10% and 18.2% respectively [12,13,17]. Abdel-Moneim et al. explained that the high rate of prevalence of HBoV-1 was reported because of a potential nosocomial pathogen among pediatric care units. This explanation was verified by Cabral et al. in (2021) after he demonstrated that bocavirus is one of the airborne respiratory conditions transmitted during the analysis of the air in pediatric emergency department waiting rooms [75]. Therefore, early diagnosis of HBoV infection in the initial hospitalization time may decrease the spread of the viral infection, especially in pediatric units [47]. Moreover, unlike other respiratory viruses, HBoV can be detected in the serum and whole blood samples of patients suffering from viremia [56].

In Egypt, Abdel-Moneim et al. (2016) studied the presence of HBoV in colorectal cancer patients and found that among one hundred and one patients, twenty-four of them (23.8%) were positive for HBoV [15]. Moreover, Niya et al. (2018) used a case versus control population to detect the presence of the HBoV genome in colorectal cancer patients’ tissue and compared the result with matched healthy control group tissue; HBoV was detected in one patient from each group, with a total prevalence of 1.3% [31].

Several studies have reported the spreading of HBoV among pilgrims during Hajj and Umrah, as mass gathering aids in the transmission of respiratory diseases. The studies concluded that raising awareness among pilgrims of the importance of following public health precautions, such as wearing masks and undergoing vaccination, significantly reduces the transmission of respiratory pathogens [57,60,70].

Currently, four genotypes have been identified worldwide (HBoV1, HBoV2, HBoV3 and HBoV4). In the MENA Region, HBoV1 is the most prominent reported genotype and is mainly associated with respiratory diseases [20,28]. However, HBoV1 was rarely detected in stool samples [25]. Genotypes 2, 3 and 4 were reported in cases of acute gastroenteritis [25,62].

HBoV is detected more frequently with other viruses in the respiratory and gastrointestinal tract (Table 2). HBoV co-infection is present at a high rate among the tested samples, especially with respiratory syncytial virus (RSV) [13,32,41], which is the most prominent virus that causes respiratory illness.

However, there is a conflict regarding the role of HBoV in cases of co-infection. Some studies reported no differences in clinical severity between patients hospitalized with a single infection (sole virus) and those with viral co-infection [13,47]. Others proved that more disease severity was associated with a high viral load detected in a single infection [18,76]. 

## 5. Conclusions

This systematic review provides a clear summary of the existing knowledge about the prevalence of HBoV infection in the MENA region. The data presented show that HBoV infection is common in children admitted to hospitals and should be screened for as a part of the standard diagnostic panels. This systematic review also highlights the importance of studying the presence of this virus alone or in association with other viruses and stresses the need for further research on the pathogenicity and genomic variation of HBoV.

## Figures and Tables

**Table 1:** The prevalence of HBoV in 14 countries of the MENA region.

Country	First Author, Year	Study Period	Age Group	HBoV Positive	Sample Size	Prevalence	Type of Specimen	HBoV Genotypes	Study Design
Egypt	Zaghloul (2011) [12]	2009–2010 (8 months)	Children (<5 years)	22	100	22.0%	NPAs	ND	Case-control
				18	100	18.0%	Serum		
	Tabl et al. (2012) [13]	2010–2011 (10 months)	Children (<12 years)	20	200	10.0%	NPAs	ND	Cross section
	EL-Mosallamy et al. (2015) [14]	2013–2015 (18 months)	Children (<2 years)	2	100	2.0%	Stool	ND	Cross section
	Abdel-Moneim et al. (2016) [15]	2011–2015	Aged between 30 and 75 years	24	101	23.8%	Colorectal cancer biopsy	Genotype 1	Cross section
	Abdel-Moneim et al. (2016) [16]	2013–2014 (2 months)	Children (<3years)	54	95	56.8%	NP	Genotype 1	Cross section
	Meligy et al. (2016) [17]	2013–2014 (5 months)	Children (<3years)	8	51	18.2%	NP and NPA	ND	Cross section
	Amr et al. (2017) [18]	2015–2016 (11 months)	Children (<5 years)	19	123	15.4%	NPAs	ND	Cross section
				16	123	13.0%	Serum		
	Hatem et al. (2019) [19]	2010–2014 (48 months)	All age groups with viral infection	1075	11	1.0%	NP and OP	ND	Cross section
	Abozahra et al. (2020) [20]	2018–2019 (5 months)	Children (<5 years)	7	75	9.3%	NP	Genotype 1	Cross section
				5	75	6.7%	Serum		
	Roshdy et al. (2020) [21]	2013–2014 (11 months)	All age groups	2	200	1.0%	NPAs	ND	Cross section
Iran	Naghipour et al. (2007) [22]	2003–2004 (4 months)	Children (<5 years)	21	261	8%	NPAs or NP	Genotype 1	Cross section
	Nadji et al. (2010) [23]	2007–2008 (11 months)	Children (<17 years)	9	133	6.8%	NPAs	Genotype 1	Cross section
		2006–2008 (32 months)		6	47	12.8%	Stool		
	Monavari et al. (2013) [24]	2010–2011 (12 months)	Children (<5 years)	16	200	8.0%	Stool	ND	Cross section
	Romani et al. (2013) [25]	2008–2010 (24 month)	All age groups	27	294	9.18%	Stool	Genotype 1, 2 and 3	Cross section
	Shokrollahi et al. (2014) [26]	2009–2011 (24 months)	Children (<9 years)	6	80	8.0%	Stool	ND	Cross section
	Mortazavi et al. (2015) [27]	2014 (5 months)	Age group between 29 and 91 years	6	91	6.6%	Throat swabs	ND	Cross section
	Tabasi et al. (2016) [28]	2012–2013 (6 months)	Children (<2 years)	15	140	10.7%	Throat swabs	Genotype 1	Cross section
	Moradi et al. (2017) [29]	2015–2016 (10 months)	Age group between 56 and 80 years	0	30	0.0%	BAL and NP	ND	Cross section
	Malekshahi et al. (2017) [30]	2013–2014 (8 months)	Children (<5 years)	0	71	0.0%	Throat swabs and nasal washes	ND	Cross section
	Niya et al. (2018) [31]	2011–2016	All age groups	1	66	1.5%	Colorectal cancer biopsy	Genotype 1	Case -control
	Mohammadi et al. (2019) [32]	2016–2017 (12 months)	Children (<3 years)	10	75	13.3%	NP	Genotype 1	Cross section
	Mohammadi et al. (2020) [33]	2017–2018 (12 months)	Children (<3 years)	67	500	13.4%	NP	ND	Cross section
				72	500	14.4%	Stool		
	Hashemi et al. (2021) [34]	NA	Confirmed COVID-19 cases from all age groups	10	105	9.7%	Throat swabs and NP	ND	Cross section
Iraq	Atyah et al. (2017) [35]	2015–2016 (8 months)	Children (<15 years)	48	195	24.6%	NP	ND	Cross section
	Al-Mayah et al. (2018) [36]	2017–2018 (2 months)	Children (<5 years)	8	122	6.6%	NP	Genotype 1	Cross section
	Shamiran et al. (2019) [37]	2017 (3 months)	Children (<5 years)	18	50	36.0%	NP and blood	ND	Cross section
	Hasan et al. (2020) [38]	2017–2018 (2 months)	Children (<5 years)	8	122	6.6%	NP	Genotype 1	Cross section
	Yaseen et al. (2020) [39]	2018–2019 (7 months)	Children (<10 years)	31	80	38.8%	Pharynx swab	ND	Cross section
				28	80	35.0%	Serum		
Israel	Hindiyeh et al. (2008) [40]	2006 (11 months)	Children (<10 years)	26	231	11.3%	Nasal suction, NP, BAL, throat swab, sputum, pleural fluid	ND	Cross section
Jordan	Kaplan et al. (2006) [41]	2003–2004 (5 months)	Children (<5 years)	57	312	18.3%	NPA	Genotype 1	Cross section
	AL-Rousan et al. (2011) [42]	2007 (11 months)	Children (<13 years)	20	220	9.1%	NPA	Genotype 1	Cross section
	Awad et al. (2020) [43]	2016 (3 months)	Children (<5 years)	12	479	2.5%	NP	ND	Cross section
Kuwait	Essa et al. (2015) [44]	2010–2013 (31 months)	All age groups	14	735	4.9%	BAL, TA, NPAs and NP	ND	Cross section
	Madi and A. AL-Adwani (2020) [45]	2018–2020 (24 months)	All age groups	111	5941	1.9%	NPAs and throat swabs	Genotype 1	Cross section
	Mohammad et al. (2020) [46]	2017 (11 months)	Children (<10 years)	2	84	2.3%	Stool	Genotype 1	Cross section
Lebanon	Finianos et al. (2016) [47]	2013–2014 (11 months)	Children (<16 years)	36	236	15.0%	NPAs	ND	Cross section
Oman	Khamis et al. (2012) [48]	2007–2008 (12 months)	Children (<5 years)	8	259	3.0%	NPAs	ND	Cross section
Qatar	Janahi et al. (2017) [49]	2010–2011 (24 months)	Children (<2 years)	15	369	4.1%	NP	ND	Cross section
	Al-Romaihi et al. (2019) [50]	2012–2017 (71 months)	Adult (>15 years)	286	37929	0.7%	OP, NP and NPAs	ND	Cross section
	Al-Romaihi et al. (2020) [51]	2012–2017 (71 months)	Children (<15 years)	1920	30946	6.2%	Throat swabs, NP and NPAs	ND	Cross section
	Nadeem et al. (2020) [52]	2013–2016	All age groups with respiratory illness	874	43106	2.0%	OP and NP	ND	Cross section
Saudi Arabia	Memish et al. (2012) [53]	2009 (6 days)	HCP age between 31 and 49 years	0	184	0.0%	Throat swabs and NP	ND	Cross section
	Abdel-Moneim et al. (2013) [54]	2012 (4 months)	Children (<10 years)	18	80	22.5%	NP	Genotype 1	Cross section
	Al-Ayed et al. (2014) [55]	2012–2013 (9 months)	Children (<5 years)	1	135	0.74%	NP	ND	Cross section
	Bubshait et al. (2015) [56]	2010–2011 (12 months)	Children with viremia (<5 years)	5	47	10.6%	Serum	ND	Cross section
	Memish et al (2015) [57]	2013 (22 day)	Pilgrims came from worldwide to do hajj or Umrah (>18 years)	2	1676	0.1%	Nasal swabs	ND	Cohort
	Eifan et al (2017) [58]	2014–2015 (11 months)	Children (<5 years)	171	2266	7.5%	NP, NPAs and BAL	ND	Cohort
	Abdel-Moneim et al. (2018) [59]	2016 (11 months)	Blood donors (adult) (20–48 years)	21	300	7.0%	Whole blood sample	Genotype 1	Cross section
	Koul et al. (2018) [60]	2014–2015 (6 MONTHS)	Pilgrims returning from Saudi Arabia, adults between 26 and 60 years	2	300	0.7%	Throat swabs and NP	ND	Cross section
Sudan	Adam et al. (2018) [61]	2014 (8 months)	Children (<5 years)	5	437	1.1%	Stool	Genotype 1	Cross section
Tunisia	Kapoor et al. (2010) [62]	NA	Children (<15 years)	32	96	33%	Stool	Genotype 1, 2,3 and 4	Case- control
	Khalifa et al. (2019) [63]	2013–2014 (15 months)	Children (<1 years)	37	515	7.2%	NPAs	ND	Cross section
Turkey	Midilli et al. (2010) [64]	NA	All age groups	7	155	4.5%	NPAs and throat swab	Genotype 1	Cross section
	Azkur et al. (2014) [65]	2011–2012 (6 months)	Children (<2 years)	3	55	5.4%	NP	ND	Cross section
	UYAR et al. (2014) [66]	2010 (6 months)	Children (<2 years)	3	62	4.8%	NPAs	ND	Case-control
	Akturk et al. (2015) [67]	2013–2014 (7months)	Children (<7 years)	30	1143	2.6%	NP	ND	Cross section
	ÇİÇEK et al. (2015) [68]	2002–2014 (151 months)	All age groups	18	5102	4%	NP, BAL and NPAs	ND	Cross section
	Demirci et al. (2016) [69]	2009 (3 months)	Children (<5 years)	8	120	6.7%	NP	ND	Cross section
	Erdem et al. (2016) [70]	2013–2014 (26 months)	Pilgrims (adult) (>15 years)	1	97	1%	NP	ND	Cross section
	Goktas et al. (2016) [71]	2014–2015 (11 months)	All age groups	91	845	10.76%	NP	ND	Cross section
	Bakir et al. (2020) [72]	2015–2017 (32 months)	Children (<18 years)	105	2310	4.5%	NP	ND	Cross section
United Arab Emirates	Alsuwaidi et al. (2018) [73]	2016-2017 (3 months)	Children (3-6 years)	0	18	0.0%	NP	ND	A pilot study
	Jeon et al. (2019) [74]	2015–2018 (27 months)	Children (<15 years)	0	198	0.0%	Sputum, NP and BAL	ND	Cross section
			Adults aged between 15 and 64 years	2	718	0.3%	Sputum, NP and BAL		
			Elderly (≥65 years)	0	446	0.0%	Sputum, NP and BAL		

NA: Not available, ND: Not detected, NPAs: Nasopharyngeal aspirates, NP: Nasopharyngeal swabs, OP: Oropharyngeal Swab, BAL: Broncho Alveolar Lavage, TA: tracheal aspirate, HCP: Health Care Provider.

**Table 2:** HBoV and other viruses detected in patients with viral co-infection.

Country	First Author, Year	Viral Coinfection Rate	Coinfected Viruses
Egypt	Tabl et al. (2012) [13]	66.7%	Respiratory syncytial virus
		13.3%	Para influenza
		6.7%	Influenza-B viruses
		6.7%	Influenza-A viruses
		6.7%	Adenovirus.
Iran	Naghipour et al. (2007) [22]	14.0%	Adenovirus
		15%	Respiratory syncytial virus
		4.0%	Influenza A virus
	Tabasi et al. (2016) [28]	13.3%	Respiratory syncytial virus
	Mohammadi et al. (2019) [32]	40%	Respiratory syncytial virus
	Mohammadi et al. (2020) [33]	65.6%	Respiratory syncytial virus
		62.5%	Rotavirus
	Hashemi et al. (2021) [34]	100.0%	Severe acute respiratory syndrome coronavirus 2
Iraq	Atyah et al. (2017) [35]	4.6%	Respiratory syncytial virus
		3.6%	Human metapneumovirus
Israel	Hindiyeh et al (2008) [40]	69.2%	Adenovirus
		7.1%	Respiratory syncytial virus
		10%	Parainfluenza virus 3
Jordan	Kaplan et al. (2006) [41]	72%	Respiratory syncytial virus
	AL-Rousan et al. (2011) [42]	20%	Respiratory syncytial virus
Kuwait	Madi and A. AL-Adwani (2020) [45]	10.8%	Respiratory syncytial virus
		9.9%	Human rhinoviruses
		6.3%	Influenza A virus
		3.6%	Adenovirus
	Mohammad et al. (2020) [46]	50%	Adenovirus
Lebanon	Finianos et al. (2016) [47]	47.2%	Adenovirus
		36.1%	Human rhinoviruses
Oman	Khamis et al. (2012) [48]	62.5%	Respiratory syncytial virus
Qatar	Janahi et al. (2017) [49]	51.2%	Respiratory syncytial virus
		25.5%	Rhinovirus
Turkey	Azkur et al. (2014) [65]	33.3%	Respiratory syncytial virus
		33.3%	Rhinovirus
		33.3%	Influenza A virus
